# Severe complicated influenza declined during the prevention of COVID-19 in Taiwan

**DOI:** 10.1017/ice.2020.272

**Published:** 2020-06-03

**Authors:** Chih-Jen Yang, Tun-Chieh Chen, Shin-Huei Kuo, Min-Han Hsieh, Yen-Hsu Chen

**Affiliations:** 1 Department of Internal Medicine, Kaohsiung Municipal Ta-Tung Hospital, Kaohsiung Medical University, Kaohsiung, Taiwan; 2 School of Medicine; 3 Graduate Institute of Medicine; 4 Center of Tropical Medicine and Infectious Diseases, Kaohsiung Medical University, Kaohsiung, Taiwan; 5 Department of Biological Science and Technology, College of Biological Science and Technology, National Chiao Tung University, Hsinchu, Taiwan

## Dear Editor

The study of face masks use in Latin America reported by *Soto* et al is quite interesting^1^. SARS-CoV2 (COVID19) is a novel coronavirus that has caused a critical public threat worldwide. How to prevent the COVID-19 outbreak is a crucial issue and community-based preparedness is important. SARS-CoV2 is transmitted through droplets and contact transmission routes, and the Taiwan government has taken many proactive and aggressive actions to prevent the transmission of COVID-19, including wearing face masks. These strategies have not only controlled COVID-19 transmission but also reduced influenza infection in Taiwan.

Although Taiwan is geographically close to China, the number of confirmed cases with COVID-19 in Taiwan is significantly lower than in other neighboring countries, with fewer than 450 confirmed COVID-19 cases and limited community transmission. The Taiwan government has acted swiftly since January to prevent the COVID-19 outbreak crisis by implementing measures such as border control, case identification and isolation, quarantine of suspected cases, extending holidays, travel restriction, education and big data integration. In addition, the export of face masks was prohibited on January 24, 2020 to avoid a shortage. In addition to these proactive early decisions, the Taiwan government acquired all available face masks and assigned most of them to hospital staff first in order to protect them from COVID-19. Moreover, mask making machine manufacturers were asked to step up face mask production capacity, which rose from 1.3 million per day to 13 million per day by the end of March 2020.

Based on the previous experience of severe acute respiratory syndrome (SARS) in 2003, the Taiwan CDC encouraged residents to wear face masks and perform hand hygiene, especially hospital staff and people in crowded places when the COVID-19 outbreak was initially reported in Wuhan, China in January 2020^2^. Recently, the Taiwan CDC announced regulations requiring all passengers using public transportation to wear face masks all the time and follow the social distancing recommendations.

**Fig. 1.**
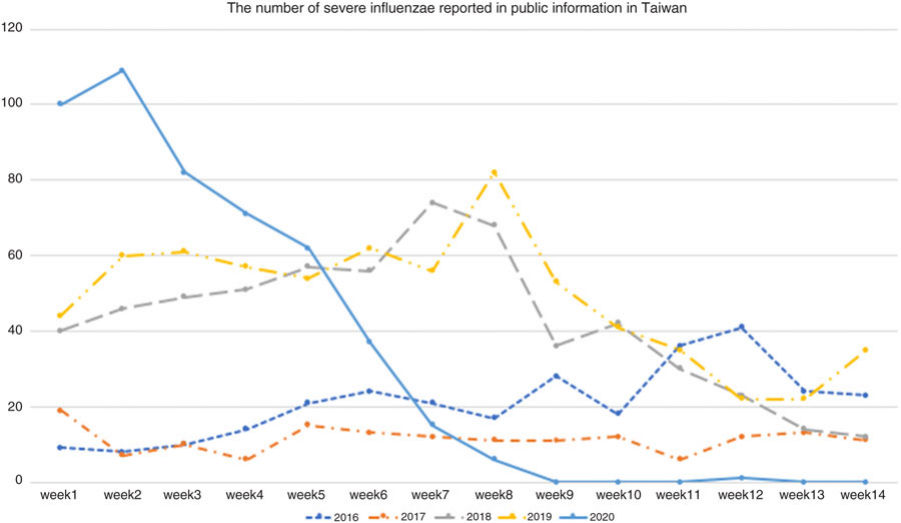
The number of cases of reported severe complicated influenza from 2016 to 2020 in Taiwan according to the Taiwan CDC (https://www.cdc.gov.tw/).

This successful policy not only controlled the community transmission of COVID-19, but also had an unexpected effect on lowering the incidence of severe complicated influenza after the sixth week this year based on the public records of the Taiwan CDC (Fig. 1). In Taiwan, the main influenza period occurs in the winter, from late November through March. Influenza cases are not required to be reported. However, patients with serious influenza complications who need intensive care unit treatment and associated deaths are classified as having a notifiable disease, “severe complicated influenza”, and these cases have to be reported within one week. Hundreds of people die of severe influenza in Taiwan every year. Since January 2020, the Taiwan CDC has required that all patients with suspected COVID-19 should also be checked for both SARS-CoV-2 and influenza virus infections. According to the Taiwan CDC, the number of cases of severe complicated influenza decreased markedly since the sixth week this year. Moreover, only one severe complicated influenza case was reported in March, compared to approximately 10 to 50 cases per month from January to March reported in the past five years. Hand hygiene and social distancing are both recommended strategies to prevent influenza based on systematic reviews^3,4^, and based on the Taiwan CDC data, we believe that a face mask wearing policy also plays an important role in the prevention influenza infection.

Face masks, when fitted properly, effectively disrupt the forward momentum of particles expelled from a cough or sneeze, preventing respiratory disease transmission^5^. In addition, a previous case report also showed that wearing a face mask helped to reduce the transmission of influenza during the COVID-19 pandemic^6^. Face masks appear to be highly efficient at filtering pathogens, including influenza and rhinoviruses^7^, and their use should be encouraged during influenza pandemics^3,8^. For healthcare workers, a meta-analysis of randomized controlled trials indicated a protective effect of masks and respirators against clinical respiratory illnesses (risk ratio [RR]: 0.59; 95% confidence interval [CI]: 0.46–0.77) and influenza-like illness (RR: 0.34; 95% CI: 0.14–0.82). Furthermore, even pooled analysis of observational studies revealed the benefit of wearing masks (odds ratio [OR] = 0.13; 95% CI: 0.03–0.62) and respirators (OR = 0.12; 95% CI: 0.06–0.26) against SARS.

Although Some studies have been against the use of face masks during the COVID-19 pandemic^9^ and one meta-analysis reported that the use of face masks to prevent transmission of influenza virus is still controversial^10^, the mask-wearing policy accompanied with hand hygiene and social distancing appear to have prevented both severe complicated influenza and COVID-19 in Taiwan. In April 2020, the Taiwan Government donated approximately 10 million face masks to countries hit hardest by SARS-COV-2, including the United States and the European Union. We hope that our experience can help other regions to overcome the COVID-19 pandemic as quickly as possible.
